# Risks and Prevention of Surgical Site Infection After Hernia Mesh Repair and the Predictive Utility of ACS-NSQIP

**DOI:** 10.1007/s11605-022-05248-6

**Published:** 2022-01-21

**Authors:** Robert Beaumont Wilson, Yasser Farooque

**Affiliations:** Department of Upper Gastro-Intestinal Surgery, Liverpool Hospital, UNSW, NSW Sydney, 2170 Australia

**Keywords:** Prevention, Surgical site infection, Antisepsis, Wound healing, Prosthesis, Hernia, Mesh, Biofilm

## Abstract

**Aim:**

The aim of this paper was to provide a narrative review of surgical site infection after hernia surgery and the influence of perioperative preventative interventions.

**Methods:**

The review was based on current national and international guidelines and a literature search.

**Results:**

Mesh infection is a highly morbid complication after hernia surgery, and is associated with hospital re-admission, increased health care costs, re-operation, hernia recurrence, impaired quality of life and plaintiff litigation. The American College of Surgeons National Surgical Quality Improvement Program is a particularly useful resource for the study and evidence-based practise of abdominal wall hernia repair.

**Discussion:**

The three major modifiable patient comorbidities significantly associated with postoperative surgical site infection in hernia surgery are obesity, tobacco smoking and diabetes mellitus. Preoperative optimization includes weight loss, cessation of smoking, and control of diabetes. Intraoperative interventions relate, in particular, to the control of fomite mediated transmission in the operating theatre and prevention of mesh contamination with *S. aureus *CFUs. Risk management strategies should also target the niche ecological conditions which enable bacterial survival and subsequent biofilm formation on an implanted mesh. Outcomes of mesh infection after hernia surgery are closely related to mesh type and porosity, patient smoking status, presence of MRSA, bacterial adhesion and biofilm production. The use of suction drains and the timing of drain removal are controversial and discussed in detail. Finally, the utility of the ACS-NSQIP Surgical Risk Calculator in predicting complications and outcomes in individual patients and the importance of quality improvement initiatives in surgical units are emphasized.

## Introduction: Mesh and Hernia Repair

**Fig. 1:**
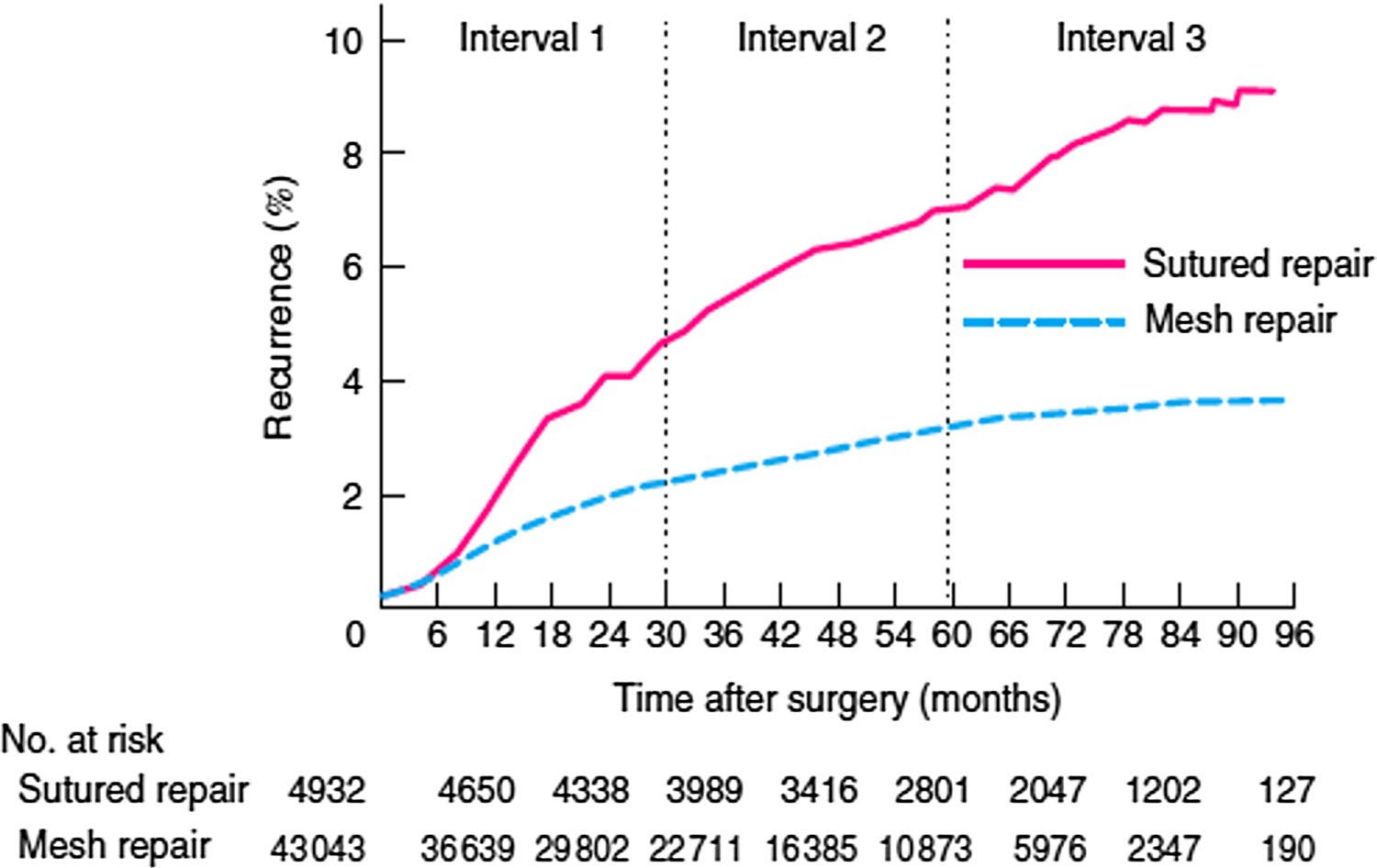
Life-table curves illustrating the risk of recurrence after sutured and Lichtenstein mesh repairs for primary inguinal hernia in men. The risk of reoperation for each interval (0–30 months, hazard ratio (HR) 0·45; 30–60 months, HR 0·38; 60–96 months, HR 0·25) (*p* < 0·001, Cox regression). Reproduced by permission^6^

The clinical use of Marlex polyethylene mesh in 1959 for incisional ventral hernia repair (VHR) and then Marlex knitted polypropylene mesh for inguinal hernia repair (IHR) ushered in a new paradigm in the repair of abdominal wall herniae.^1,2^ The Lichtenstein mesh repair for inguinal hernia and modified Stoppa-Rives preperitoneal and retro-rectus mesh repairs subsequently became the standard in hernia surgery. Mesh reinforcement allowed tension-free repairs, improved perioperative pain and shortened hospital stay, and was associated with decreased long-term hernia recurrence rates.^3^ For example, in a 2014 pooled analysis of 637 sutured versus 1145 synthetic mesh repairs of primary ventral hernia, the respective recurrence rate was 8.2% vs 2.7% (log OR, − 1.05; 95% CI, − 1.58 to − 0.52; *p* < 0.001).^4^ A 2016 meta-analysis of randomized controlled trials (RCT) of sutured versus mesh repair of incisional and primary ventral hernia found a significant reduction in hernia recurrence with the use of mesh (relative risk (RR) = 0.36; 95% CI, 0.27 to 0.49; *p* < 0.00001).^5^ The risk of re-operation for recurrence after Lichtenstein repair was 25% of that of sutured repair in an analysis of the Danish Hernia Database involving 47,975 male patients 5 years or more after primary IHR (Cox hazard ratio (HR) = 0.25 (95% CI, 0·16 to 0·40; *p* < 0.001). Sutured repairs in this series included McVay, Shouldice, annulorrhaphy or Bassini repair (Fig. 1).^6^

## Mesh and SSI

Whilst non-absorbable synthetic mesh implantation improved the observed hernia recurrence rates, it was also associated with increased wound complications in open VHR and IHR, including seroma formation, foreign body reaction, mesh migration, adhesions, chronic pain and surgical site infection (SSI).^7–10^ An SSI is an infection occurring at the surgical site up to 30 days after surgery or up to 12 months after prosthetic implant surgery. The American College of Surgeons National Surgical Quality Improvement Program (NSQIP) criteria for diagnosis of SSI are summarized in Table 1. ^10^The 3 categories of SSIs as defined by the US National Healthcare Safety Network (NHSN)/Centres for Disease Control (CDC) are as follows:

1. superficial incisional (involves only skin and subcutaneous tissue of the incision)
2. deep incisional (involves deep soft tissues of the incision, for example fascial and muscle layers)
3. organ/body space (involves any part of the body deeper than the fascial/muscle layers, for example peritoneal cavity, viscera).^11,12^

**Table 1:** Clinical findings that confirm the diagnosis of an SSI. Reproduced by permission^10^

Purulent drainage
Cardinal sign of infection (rubor, calor, tumour, dolor, functio laesa)
Documentation for SSI by surgeon or other attending physician
Deliberate opening of incision by surgeon (unless culture negative)
Organism isolated by aseptically acquired culture
Abscess or other evidence of infection during examination, re-operation or histology

SSI is the most common reason for re-admission to hospital after surgery in the USA. In patients who develop SSI after hernia surgery, up to 80% will re-present after they have been discharged from hospital.^12^ After hernia mesh repair, deep incisional or organ space SSIs which involve the mesh should be distinguished from superficial incisional SSIs. The implementation of evidence-based protocols can potentially prevent up to half of all SSIs.^13,14^ Up to 99% of elective hernia repairs are clean (class I) or clean-contaminated (class II) wound classification (Table 2).^9,11^

**Table 2:** CDC classification of wound classes^11^

Class 1:	Clean wounds No infection, no inflammation, primarily closed No entry of respiratory, alimentary, genital or urinary tracts If wound drainage is necessary, closed suction drainage is required *Example:* Simple elective hernia repair
Class II:	Clean-contaminated wounds Entry into respiratory, alimentary, genital or urinary tracts under controlled conditions *Example:* Elective hernia repair with cholecystectomy
Class III:	Contaminated wounds Fresh, open wounds with break in sterile technique, leakage of gastrointestinal contents into the wound or contact with acute, non-purulent inflammation *Example:* Hernia repair with enterotomy and leakage of enteric contents into wound
Class IV:	Dirty wounds Old traumatic wounds with devitalized tissue and those that involve existing clinical infection or perforated viscus *Example:* Removal of infected hernia mesh

Mesh infection is a highly morbid complication after hernia surgery. It is associated with hospital re-admission, increased healthcare costs, re-operation, hernia recurrence, impaired quality of life and plaintiff litigation.^8^ The overall rate of hernia mesh infection ranges from 1 to 8% in various series.^8^ This is related to pre-existing patient comorbidities, surgical technique, mesh selection and infection risk prevention strategies in individual institutions. The onset of mesh infection is associated with a contaminated surgical field, a prolonged operation time or early wound complications.^15^ Open ventral hernia mesh repairs have higher reported mesh infection rates (6–10%) than laparoscopic hernia repairs (0–3.6%).^16^ Patients with large, complex ventral or inguinal herniae requiring open surgery who have multiple comorbidities (advanced age, American Society of Anaesthesiologists (ASA) score ≥ 3, malnutrition, diabetes, immunosuppression, tobacco smoking or obesity (BMI ≥ 35 kg/m^2^)) are particularly at risk. Despite the published rates of SSI in clean surgery being historically < 2%, the SSI rate in clean open inguinal hernia surgery varies from 2.4 to 4.9%.^17,18^ The average cost in the USA per SSI event is US$11,000, whilst mesh infection cost over US$75,000 and enterocutaneous fistula greater than US$200,000 per event.^19^ Strict risk reduction protocols including the control of patient comorbidities prior to elective hernia surgery may result in substantial cost savings.^20^

## Properties of Mesh

The mechanical and biological properties of different meshes influence their handling behaviour, tissue incorporation, biocompatibility, infection risk and need for explantation. These properties include non-absorbable synthetic polymer composition (polypropylene, polyester, expanded polytetrafluoroethylene (ePTFE)), absorbable synthetic polymer composition (polyglycolic acid (PGA), polylactide (PLA), polycaprolactone (PCL), polydioxanone (PDO), poly-4-hydroxybutyrate (P-4HB)), biological (human, bovine or porcine derived acellular collagen), fabric construction (woven or knitted), fibre type (monofilament or multifilament), filament diameter and pore size (macroporous or microporous).^8,21^ A mesh porosity of > 60% is used to further segregate mesh textile types into class 1 mesh (macroporous) and class II mesh (macroporous with micropores). Textile mesh porosity is closely related to the interfilament distance and mesh weight. In polypropylene meshes, a minimum interfilament distance of 1000 μm is necessary to prevent bridging of scar tissue across the entire pore. This is important in the prevention of scar plate formation, mesh contraction and chronic pain after hernia surgery. Such interfilament distances are found in class I mesh but not class II mesh (Table 3).^22^

**Table 3:** Explanted mesh samples and assignment to mesh class. Reproduced by permission^22^

Brand name	Weight (g/m^2^)	Textile porosity (%)	Mesh class
Vypro	38	77	1
Ultrapro	28	67	1
Ti-mesh	35	68	1
Mersilene	40	71	1
Marlex	95	37	2
Prolene	109	56	2
Atrium	90	50	2
Surgipro	87	65	2*
ePTFE	400	0	4

Textile porosity reflects in a two-dimensional image the area that is not covered by the filaments; measurements were provided by the manufacturer

^*^However, both monofilament and multifilament Surgipro meshes showed rather small pores, and as we microscopically could never see interfilament distances of more than 500 μm at explanted Surgipro meshes, we considered this mesh as small pore construction, though information provided by the manufacturers indicated a textile porosity of 65%

The amount of foreign material remaining in the hernia wound after mesh repair is related to the mesh type, porosity and weight. This determines whether mesh will be eventually incorporated, encapsulated or degraded. There are 4 groupings of simple, composite or combined meshes based on their mesh weight.^22^

1. Ultralight ≤ 35 g/m^2^
2. Light C 35–70 g/m^2^
3. Standard C 70–140 g/m^2^
4. Heavy C ≥ 140 g/m^2^

Macroporous mesh is associated with increased adhesion formation and erosions, whereas microporous mesh is more prone to mesh infections, encapsulation, shrinkage and seromas.^8^ A classification system of hernia mesh was proposed in 2012:

- Class I: macroporous
- Class II: macroporous with micropores
- Class III: microporous (porous mesh with anti-adhesive films)
- Class IV: submicronic pore size, film like mesh without porosity
- Class V: 3 dimensional mesh/ plugs
- Class VI: biological mesh
- Class VIc: synthetic absorbable mesh.^22^

In an analysis of 1000 explanted mesh cases, mesh removal (for pain or mesh infection) was significantly over-represented by class II and V mesh, and removal due to hernia recurrence was significantly over-represented by lightweight class I mesh.^22^ The composite meshes (class III) may have decreased *effective* porosity due to anti-adhesion films or mesh construction.^23^

Subsequent to these studies, laparoscopic repair using a bridging monofilament lightweight polyester mesh with a hydrophilic porcine dermis collagen barrier (Parietex Composite Optimised Mesh, Covidien, New Haven, CT, USA) was found to have a late (5 year) recurrence rate of 9.4%. It was thought recurrences were related to loss of elasticity and tensile strength, mesh degradation and stretching. Over time, the intra-abdominal forces that are generated by straining or coughing are sufficient to cause mesh failure in the absence of primary tissue reinforcement. This has led to the selection of composite meshes which still maintain suitable porosity characteristics, suture retention strength (> 20 N), burst strength (> 50 N/cm), strain at 16 N/cm (10–30%) and resistance to tearing (> 20 N) for use in VHR.^21,24,25^ Such mesh properties are particularly important in obese males who may generate very high tensile stress forces within the abdomen of 47.8 N/cm at an intra-abdominal pressure (IAP) of 30 kPa, or in very large ventral hernial defects without primary fascial closure.^21^

## Microbiology of Mesh Infection

The most common bacteria associated with prosthetic mesh infection are *Staphylococcus aureus* (57.7%), of which up to half are methicillin-resistant *Staphylococcus aureus* (MRSA). Other bacterial species include *Staphylococcus epidermidis*, *Enterococcus faecalis*, Gram-negative bacteria (26.1%) (*Escherichia coli*, *Klebsiella* spp., *Pseudomonas aeruginosa*, *Enterobacter cloacae*) and Gram-positive anaerobic cocci (*Peptostreptococcus*, *Finegoldia* spp.).^8,18,26^ One study found in 63% of postoperative incisional hernia mesh repair infections, the causative organism was MRSA.^8^ Bacteria attach more readily to non-polar, hydrophobic surfaces including PTFE and polypropylene than to hydrophilic surfaces such as metals or glass.^27^ The initial period of bacterial adhesion can be rapid and reversible. However, subsequent irreversible mesh attachment via bacterial adhesins and production of bacterial biofilm impairs penetration and clearance of bacteria by host immune cells and systemic antibiotics.^28^

The life cycle of a bacterial biofilm involves 4 phases: adhesion, proliferation/accumulation, maturation and detachment/dispersal. Development and maturation of a biofilm can occur within 10 h of wound contamination. Haematoma or extracellular matrix proteins (fibrinogen, elastin, collagen, fibronectin) on a medical prosthesis such as hernia mesh provide a substrate for Staphylococcal bacterial adhesion via microbial surface components recognizing adhesive matrix molecules (MSCRAMMs).^28–33^ Biofilm producing organisms such as *S. epidermidis* have a very strong affinity for fibrinogen via a “dock, lock and latch” mechanism.^28^ After adhesion to a prosthetic surface, planktonic bacteria transition to an exponential growth phase, with bacterial production of extracellular polymeric substance (EPS). EPS is a complex matrix comprised of proteins, exopolysaccharides (polysaccharide intercellular adhesin (PIA)), glycerol teichoic acid and extracellular DNA (eDNA). PIA is important during the accumulation phase for bacterial intercellular attachment and protection of the biofilm from shear forces. Biofilm maturation occurs with formation of a cross-linked 3-dimensional matrix with encased bacterial colonies, and growth then slows. Planktonic bacteria (free floating) or clusters (flocs) can later detach to create new infections or biofilms elsewhere, leading to acute infectious exacerbations in individual patients.^28^ These can include wound cellulitis, abscess formation, sinus discharge and enterocutaneous fistula.^26^ In between these acute episodes of sepsis, mesh biofilm infections are characterized by low-grade, chronic inflammation with minimal suppuration, which can lead to mesh fibrosis and contraction, chronic pain or even mesh mechanical failure.^23^

Mesh biofilms can harbour a range of different bacteria including aerobic and anaerobic species, which may not be identified by standard culture techniques in the microbiology laboratory.^23,26^ Using sonication, confocal laser scanning microscopy (CLSM), Ibis T5000 and fluorescent in situ hybridization (FISH) techniques, it was shown that biofilms are often polymicrobial with considerable heterogeneity on infected hernia mesh, even in individual patients.^23,26^ There may be cooperative relationships and communication between *S. aureus* and other organisms (*Enterococcus faecalis*, *Enterobacter cloacae*, *E.coli*, *Candida* spp.) in a polymicrobial biofilm.^26,30^ For example, horizontal gene transfer between different bacterial species is more likely to occur in a polymicrobial biofilm, including vancomycin resistance acquisition by *S. aureus* from vancomycin-resistant *E. faecalis* (VRE) via plasmid transfer.^31^

*Staphylococcus aureus* biofilms are multilayered, with heterogeneous protein expression and environmental conditions throughout. Staphylococci in the biofilm are found in four distinct metabolic states: aerobic, fermentative, dormant or dead.^30^ Dormant bacteria embedded in the mature biofilm matrix are anoxic and nutrient deprived, with associated low metabolic rates and decreased ATP production. As such, these quiescent bacteria are markedly resistant to bactericidal antibiotics (10–1000-fold), in comparison to the metabolically active, aerobic planktonic bacteria at the biofilm surface interface with oxygenated tissues.^30^ Decreased mitochondrial respiration in dormant bacteria or cytoplasmic fermentative metabolism contribute to lowered transmembrane electrical potential and proton motive force. This can inhibit the influx of cationic molecules such as aminoglycoside antibiotics.^31^ In addition, MRSA bacteria are more likely to carry genes for aminoglycoside resistance than methicillin-sensitive *S. aureus* (MSSA).

**Fig. 2:**
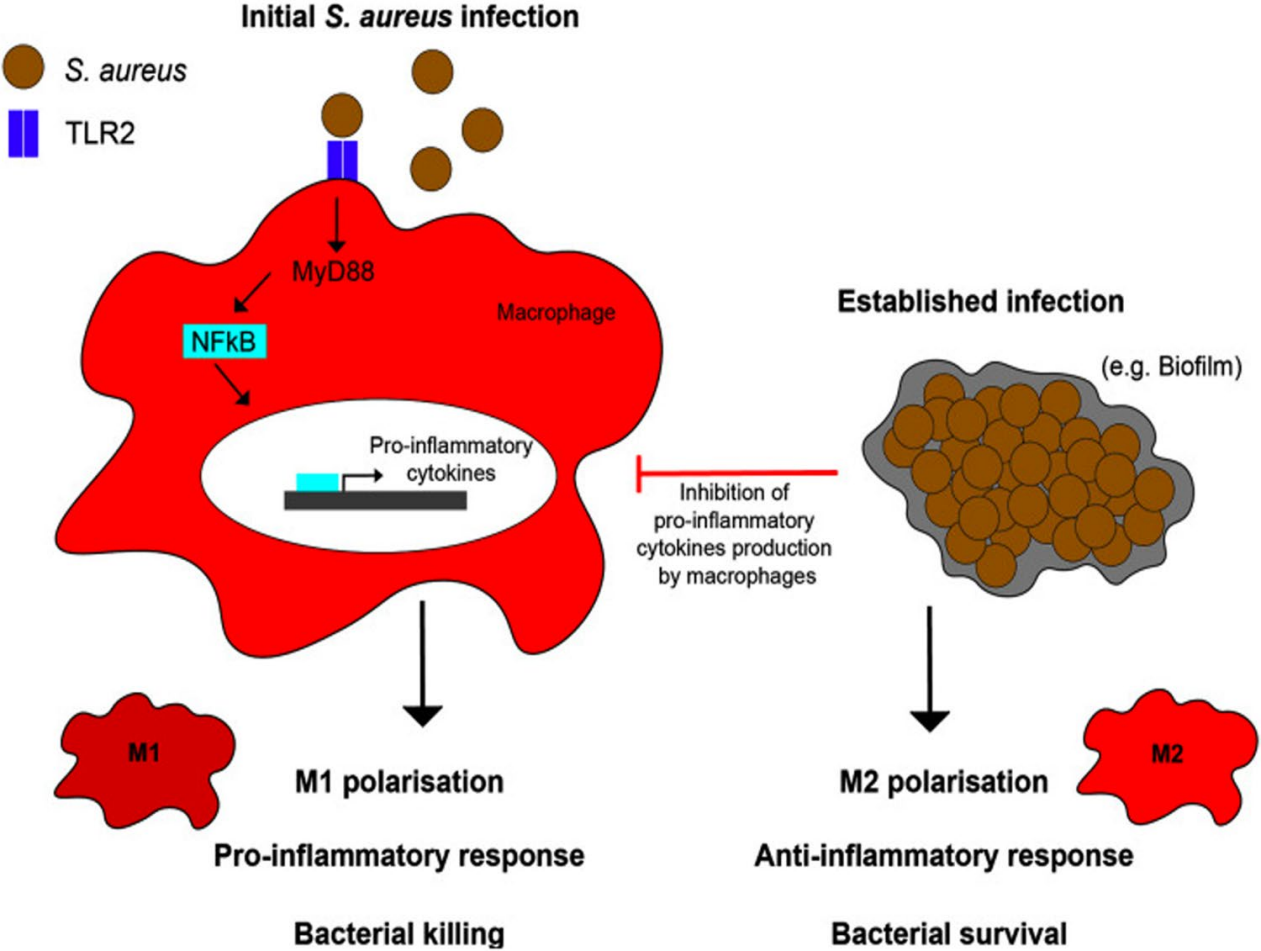
Macrophage polarization responses to *S. aureus*. M1 polarization in response to planktonic (or initial) infections occurs through Toll-like receptor 2 (TLR2), Myeloid differentiation factor 88 (MyD88) and Nuclear factor kappa-light-chain-enhancer of activated B cells (NF-kB signaling) resulting in a pro-inflammatory phenotype and cytokine production. In comparison, M2 polarization in response to established infections, such as biofilms, occurs through inhibition of macrophage pro-inflammatory cytokine production. Reproduced by permission^29^

*Staphylococcus aureus* and *S. epidermidis* biofilms provide a physical barrier for penetration of antibiotics, including oxacillin, cefotaxime and vancomycin.^30^ Rifampicin is effective against dormant *S. aureus* in a biofilm, but should be administered in combination with vancomycin or a fluoroquinolone to prevent rapid emergence of resistance. Defouling or antiseptic agents containing reactive chlorine species (hypochlorite, chloramines, chlorine dioxide) or hydrogen peroxide can be deactivated in the outer layers of the biofilm.^30^ *Staphylococcus aureus* biofilms do not stimulate release of proinflammatory chemokines (monocyte chemotactic protein-1 (MCP-1/CCL2) and Chemokine (C-X-C motif) ligand 2 (CXCL2)) or cytokines (TNF-α, IL-1β), which normally recruit and activate host macrophages and neutrophils in response to planktonic bacteria. Thus, resident macrophages are not polarized to an M1 bactericidal phenotype but rather an anti-inflammatory, profibrotic M2 phenotype in the presence of mature biofilms. This contributes to the altered host immune response in *S. aureus* mesh infections with impaired complement fixation, opsonization, phagocytosis and clearance of bacteria^23,29,30^ (Fig. 2).

The effective porosity of a mesh influences the risk of biofilm development.^23^ Microporous mesh with a pore size of < 10 μm allows ingress of bacteria (0.2- to 5-μm long) but not host polymorphonuclear leucocytes (12–15-μm diameter), or macrophages (21 μm). This means that microporous mesh and submicronic porous mesh (ePTFE) are particularly prone to bacterial biofilm formation. Such meshes do not respond to conservative management such as intravenous antibiotics, mechanical debridement and wound drainage, and will usually require re-operation and mesh explantation.^8^ Multifilament polyester mesh may also be more susceptible to bacterial colonization and infection than monofilament polypropylene mesh.^9,11^ The incidence of infection with the use of monofilament polyester mesh is similar to monofilament polypropylene mesh infection rates.^10^ Successful salvage of infected mesh has been reported in up to 55% of cases by re-operation, debridement, antibiotic treatment and negative pressure devices such as VAC®.^34^ Surgical debridement can disrupt the biofilm and expose planktonic bacteria, which are antibiotic sensitive. However, reformation and maturation of a biofilm can occur within 3 days of debridement.^32^ Re-operation for removal of infected mesh has been reported from weeks to 10 years after hernia mesh repair surgery.^15^

In longer term follow-up, the overall successful salvage rate of infected mesh without further episodes of sepsis may be as low as 10%.^34^ This is particularly determined by the mesh type, mesh porosity, presence of MRSA infection or current smoking status of the patient. In a series of 161 cases of infected hernia mesh with mean follow-up of 33.9 months, no patients were successfully salvaged who had infected polyester or composite mesh, with a salvage rate of 19.6% of polypropylene mesh versus 4.5% in PTFE. Infected lightweight polypropylene mesh was more likely to be salvaged than infected mid- or heavyweight polypropylene mesh (62.5% vs 12.5%). Infected PTFE or multifilament polyester meshes do not respond to negative pressure therapy due to persistent bacterial biofilm and the lack of formation of vascularized granulation tissue, which is required for proper mesh incorporation and bacterial clearance.^34^

## Absorbable vs Permanent Mesh

Complex abdominal wall herniae include those involving class III or IV surgical wounds, open abdomen, hernia mesh infection, strangulated hernia with bowel resection, parastomal hernia, enterocutaneous fistula or large abdominal wall hernia (≥ 10 cm in width). These provide challenges in terms of timing of surgery, choice of repair and selection of mesh.^9,11^ The use of absorbable synthetic (polyglactin 910, (Vicryl: Ethicon Inc., Somerville, NJ)) or biological (acellular dermal matrix collagen) mesh in clean or contaminated wounds may be associated with less infection risk but higher rates of recurrent hernia.^35^ Risk of hernia recurrence is increased in *bridging* VHR where no fascial closure or component separation is used, and a *chimney effect* of mesh eventration may be created in the fascial defect.^15,36^ The choice of permanent synthetic mesh over biologic or biosynthetic mesh in a contaminated/dirty wound, or as a replacement mesh in an infected hernia wound is controversial.^34,37^ Indeed, the ability of biological mesh to resist infection as compared to synthetic mesh was recently challenged in an *in vitro* experiment investigating inoculation of mesh with a single species of MRSA. It was found human dermal collagen mesh (Bard® Davol Inc., Cranston, RI) was significantly more prone to develop larger and more extensive MRSA biofilms with greater substratum penetration than absorbable synthetic polyglactin 910 woven mesh, or permanent synthetic polypropylene mesh (Bard® Davol Inc., Cranston, RI). Bacterial adhesion and biofilm formation were thought to be related in part to differences in mesh porosity, hydrophobicity and filament number.^38^ This raises the question of the suitability and cost-effectiveness of using biological mesh instead of macroporous polypropylene mesh in both clean or contaminated ventral hernia surgery.^14,39–41^ Poly-4-hydroxybutyrate monofilament biosynthetic mesh in clean or contaminated VHR appears a reliable and cost-effective long-term alternative to biological or non-absorbable synthetic mesh, but its use is still being evaluated.^42^

**Fig. 3:**
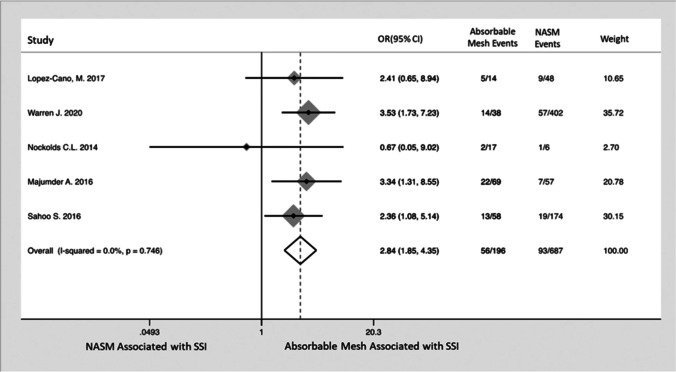
Forest plot comparing odds ratio (OR) of SSI after VHR using non-absorbable synthetic mesh (NASM) or absorbable mesh (including biosynthetic and biological mesh) in a contaminated field. Overall, the use of absorbable mesh was associated with a 2.84 increased OR (95% CI) of SSI. Reproduced by permission^41^

A synchronous hernia mesh repair should not be contraindicated during other intra-abdominal operations (appendicectomy, cholecystectomy, small bowel resection).^8,9,11^ An extraperitoneal mesh repair (retrorectus or onlay) rather than intraperitoneal mesh repair is preferable in contaminated VHR, emergency VHR, emergency laparotomy or with concurrent colorectal surgery.^9,11,40,41^ Some surgical societies including the WSES and the Ventral Hernia Working Group advocate the use of simple suture or biological mesh in contaminated/dirty VHR cases. However, this approach has not been shown to be superior to macroporous polypropylene VHR with respect to SSI, surgical site occurrence (SSO), unplanned re-operation, cost or hernia recurrence in recent systematic reviews^11,37,40,41^ (Fig. 3). This is important when resources are limited, as the cost of a single biological mesh can be equivalent to 100 permanent synthetic meshes.^37^

## Sources of Infection

Intraoperative mesh infection can occur from endogenous (patient-derived microbiota) or exogenous sources (operating theatre environment). Although the contribution of endogenous skin microbiota to SSIs after hernia surgery may be relatively small, there are some patient subgroups who are more susceptible.^33,43^ Nasal carriage of *S. aureus* is a risk factor for nosocomial infections, particularly in orthopaedic and cardiac surgery. However, the contribution of pre-operative MRSA skin or nares colonization to postoperative MRSA SSI in VHR surgery is still being investigated. Pre-operative *S. aureus* decolonization (4% chlorhexidine daily total body wash, topical 2% nasal mupirocin ointment bd for 5 days) was recommended in the WHO guidelines for known *S. aureus* carriers having prosthetic implant surgery.^10,44,45^

Contaminated airborne particles are the source of most SSIs in clean surgery.^33^ This is closely related to the number of staff and the amount of traffic in the operating theatre during a prosthetic implant. There are up to 5.6 million particles/m^3^ in a ventilated operating theatre during surgery. Such particles comprise single bacteria, clusters of bacteria (colony-forming units (CFU)) or bacteria-laden carrier particles such as respiratory droplets, lint and skin scales.^46^ These can fall into the wound from the theatre atmosphere (30%) or be transferred into the wound from the surgeon’s gloves or surgical instruments (70%).^33^ Contamination of the wound with > 10^5^ CFU/g significantly increases the risk of postoperative SSI.^10^ However, contamination with as little as 10^2^ CFU/g of *S. aureus* is sufficient to produce an infection when foreign material is present at the surgical site, related to bacterial adhesion and biofilm formation on the prosthetic surface.^10^

Skin scales or squames range from 5 to 500 μm in diameter, and can readily pass through tightly woven cloth materials. They are shed from the patient or operating team member at a rate of 7000 particles/min, and often carry viable bacteria including *S. aureus*. Particle dispersal may be worsened by turbulent air flow or external forced air warming devices.^47,48^ Interventions to reduce airborne particulate dispersal during surgery include high efficiency particulate air (HEPA)-filtered unidirectional theatre ventilation and CO_2_ diffuser insufflation of open wounds, restriction of foot traffic and staff numbers in each operating theatre, and antiseptic cleaning of theatre surfaces and portable electronic devices. HEPA filters are 99.97% efficient in removing particles ≥ 0.3 μm in diameter from theatre air ventilation. The effectiveness of laminar air flow in operating theatres in the prevention of SSIs remains controversial.^46,49^

## Controllable Risk Factors for SSI

The implementation of evidence-based protocols to prevent SSIs can substantially decrease the incidence of SSIs and healthcare costs after abdominal hernia surgery. These include CDC, NICE and WHO guidelines for the prevention of SSIs, Enhanced Recovery After Surgery (ERAS) protocols and Perioperative Quality Initiatives.^13,33,50,51^

These are based on systematic reviews and consensus statements and relate to:

1. Control of contaminated airborne particles in the operating theatre (air delivery and filtration systems, mandated air changes, positive pressure theatre ventilation, operating theatre discipline, reduction of foot traffic, light positioning, hand hygiene, operating theatre attire, theatre cleaning).
2. Patient selection and preparation (control of patient comorbidities, patient prehabilitation, MRSA decolonization, pre-operative soap/antiseptic body wash, avoidance of skin shaving, chlorhexidine gluconate 2% in isopropyl 70% alcohol antiseptic solution for preparation of the operative skin site, prevention of perioperative hypothermia/hypoxia, perioperative glycaemic control, proper selection, dosage and timing of administration of systemic antibiotic prophylaxis prior to surgical incision).
3. Surgical technique/heuristics (choice and timing of surgery, length of operation, open versus laparoscopic hernia repair, minimal handling/trauma of tissues, aseptic practice, careful haemostasis, minimization of large skin flaps/seroma formation, wound closure and drainage, optimal timing of drain removal, external negative pressure dressings, occlusive wound dressings).

Implementation of evidence-based guidelines includes patient selection for elective hernia repair, pre-operative risk interventions, improved operative technique and centralization of complex hernia surgery. This is related to modifiable comorbidities (MCM) which increase the incidence of SSI, including tobacco smoking, obesity and diabetes mellitus. A quality improvement initiative at a single-centre safety-net US academic institution included establishment of a complex hernia specialist unit and the implementation of 6 evidence-based interventions:

1. Elective VHR is not recommended for patients with BMI ≥ 35 kg/m^2^
2. Elective VHR is not recommended for current smokers.
3. Elective VHR is not recommended for patients with a haemoglobin A1c ≥ 8.0%.
4. Patients with BMI 30–50 kg/m^2^ or serum haemoglobin A1c 6.5–8.0% require individualized interventions to reduce surgical risk.
5. Mesh reinforcement is recommended for elective VHR with no contamination.
6. Laparoscopic repair is recommended for clean elective VHR.

Patients in the postquality improvement period had significantly reduced SSI rates compared to historical controls (13.5% vs. 1.5%; *p* < 0.001).^14^ Laparoscopic repair is particularly useful in reducing SSI rates in obese patients by decreasing the size and surgical manipulation of the wound, changing the proximity of the mesh to the incision, minimizing mesh contamination and maintaining immune function as compared to open VHR surgery. However, hernia recurrence rates in either primary or incisional VHR are not reduced by a laparoscopic approach as compared to open surgery.^19^

## NSQIP and Risk of Smoking

**Fig. 4:**
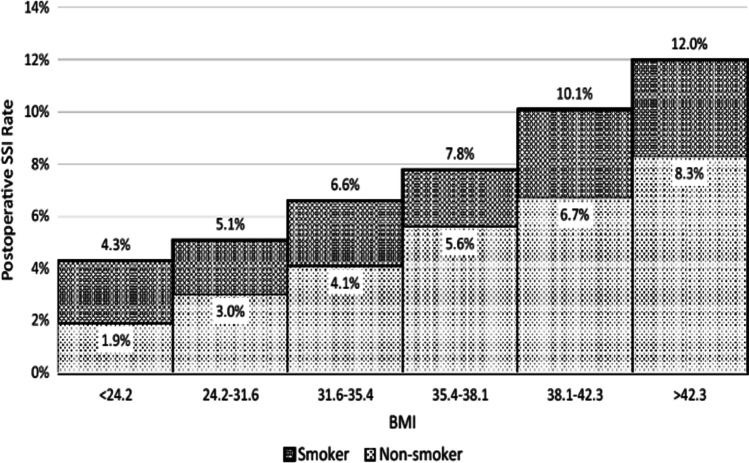
SSI rates after open ventral hernia repair within BMI groups; smokers vs. non-smokers. Reproduced by permission^52^

Smokers have higher rates of SSI and recurrent hernia after VHR or IHR than non-smokers.^52,53^ From the ACS-NSQIP database of 55,240 patients who had elective, open VHR between 2011 and 2016, 2620 (4.7%) developed SSIs (superficial: 58.5%, deep: 27%, organ-space: 16%). The lowest SSI rate (1.9%) was found in non-smokers with a BMI < 24.2 kg/m^2^. The rate of SSI increased in a stepwise fashion as the BMI rose from 24.2 to > 42.3 kg/m^2^. This was augmented by tobacco smoking, such that smokers with a BMI > 42.3 kg/m^2^ had the highest rate of SSI (12%) (Fig. 4).^52^ Smokers are more likely to have tissue hypoxia, neutrophilia, activated neutrophil collagenase (MMP-8, MMP-9), elevated carboxyhaemoglobin, comorbid chronic obstructive pulmonary disease (COPD), diabetes, cardiovascular disease, poor wound healing, nutritional and vitamin C deficiency.^52,53^ Vitamin C is an essential co-factor for collagen synthesis, neutrophil function (chemotaxis, phagocytosis, bactericidal oxidative burst), production of vasopressin and noradrenaline and anti-oxidant protection. Smokers have lower vitamin C levels compared to non-smokers. This is related to inadequate oral vitamin C intake and greater systemic utilization of vitamin C due to increased oxidative stress in smokers.^54,55^

From the ACS-NSQIP database of 220,629 patients who underwent elective hernia repair between 2011 and 2014, 40,446 (18.3%) were self-reported cigarette smokers within the past 12 months. Smoking status was based on history provided by the patient rather than laboratory testing, and thus the overall percentage of smokers may be an underestimate. Elective hernia repairs included open or laparoscopic inguinal, ventral, umbilical or incisional. A multivariable logistic regression model was adjusted for sex, age, race, BMI, hernia repair type, ASA class and related comorbidities including COPD, diabetes and hypertension. The smoking cohort had a significantly higher likelihood (95% CI) of death (OR 1.53), SSI (superficial OR 1.34, deep OR 1.31, organ space OR 1.45), pneumonia (OR 2.30), re-intubation (OR 1.82), septic shock (OR 1.31), AMI (OR 1.27), return to operating theatre (OR 1.23), hospital re-admission (OR 1.24) and wound dehiscence (OR 1.41) than non-smokers.^56^

## Cessation of Smoking

Cessation of smoking for 4 weeks improves plasma vitamin C levels, procollagen I N-propeptide (PINP) production and significantly reduces postoperative complication rates including SSI in hernia repair.^57–61^ The recovery of macrophage and neutrophil function and oxidative bactericidal mechanisms after smoking cessation is more rapid than that of wound proliferation and remodeling mechanisms (epidermal regeneration, fibroblast proliferation, collagen synthesis and deposition).^61^ In an experimental model of smoking cessation utilizing transdermal nicotine patches, abstinence from smoking for 4 weeks reduced incisional wound infections to a level similar to never smokers. The incisional wound infection rate was 12% in smokers and 2% in never smokers (*p* < 0.05). However, the wound dehiscence rate (12%) was the same in continuous smokers and abstinent smokers at 4, 8 or 12 weeks as compared to zero in never smokers.^57^ Nicotine replacement therapy (NRT) increases the rate of smoking cessation by 50–70%, and its effectiveness appears to be independent of the intensity of smoking cessation support. NRT does not appear to have detrimental effects on postoperative wound healing.^61^

## Modifiable Comorbidities and SSI

The risk of SSI after elective open incisional hernia repair is significantly higher in patients with modifiable comorbidities (smoking, obesity, diabetes mellitus). On multivariate analysis of 3908 patients who had open, elective, incisional hernia repair with permanent synthetic mesh in clean wounds from the Americas Hernia Society Quality Collaborative (AHSQC) registry, the likelihood of postoperative SSI rose with increasing combinations of MCM: patients with diabetes (OR 1.6), obese diabetics (OR 2.0) or all three MCM (OR 2.4).^62^ Obesity is associated with poor wound healing and increased SSOs, pre-existing micronutrient deficiencies, larger hernia size (> 10-cm transverse width), thick subcutaneous fat, open surgery, more extensive dissection, longer operating times, wound drains, greater bleeding, dead space and risk of wound inoculation with bacteria, and decreased peri-operative subcutaneous tissue oxygenation.^63^ Increasing BMI is also associated with an increased risk of hernia recurrence in long-term follow-up after VHR, with recurrences of 30–40% in obesity classes I–II (BMI = 30–40 kg/m^2^), and 30–50% in obesity class III (BMI > 40 kg/m^2^). A small RCT of weight loss (≥ 7% of TBW) prior to elective incisional VHR resulted in decreased complication rates and improved the likelihood of patients being hernia free.^64^ Pre-operative weight loss interventions as part of prehabilitation include medical interventions (cognitive behaviour therapy, structured exercise and dietary programmes), medications (metformin, synthetic GLP-1 receptor agonists, phentermine/topiramate, bupropion/naltrexone) or referral to a bariatric surgical service. Bariatric surgery not only improves morbid obesity, hypertension, obstructive sleep apnoea, body composition, mobility and functional status, but can also lead to resolution of diabetes prior to abdominal wall reconstruction.^65^

## Diabetes and SSI

Diabetes mellitus is a risk factor for postoperative SSI because of its close association with morbid obesity, but also a hyperglycaemic environment. Elevated levels of glucose and glycation in the blood, tissues and cells impair T cell-mediated immunity, polymorphonuclear leucocyte function (chemotaxis, diapedesis, bacterial phagocytosis and lysis), complement activation and cytokine response. Glycation can inhibit T lymphocyte production of interferon gamma (IFN-γ) and tumour necrosis factor (TNF)-α, as well as production of IL-10 by myeloid cells. This enables bacteria to evade host immune-surveillance and more readily adhere, proliferate and form biofilms in diabetic patients.^66^ Because of this effect of hyperglycaemia and glycation on cell-mediated immunity, diabetic patients are 7.25 times more likely to develop postoperative SSI than non-diabetic patients, and patients with poorly controlled diabetes mellitus are 3.25 times more likely to develop an SSI than controlled diabetic patients. From the NSQIP database, 25,819 of 219,625 patients who underwent VHR between 2005 and 2012 had diabetes. In open VHR, patients with diabetes mellitus had an increased complication rate (*p* < 0.0001) compared to non-diabetic patients, some of which was related to diabetic patients being older, more obese and with higher comorbidities (renal and cardiopulmonary). On multivariate analysis of open VHR, patients with insulin-dependent diabetes had further significantly increased odds of wound complications (wound disruption, superficial and deep SSI (OR: 1.42)) and major complications (OR: 1.73).^67^

Interventions to control pre-operative glycation levels and perioperative hyperglycaemia, as well as performing laparoscopic instead of open VHR when possible, are advocated to improve outcomes in diabetic patients. Diabetic patients with pre-operative glycated haemoglobin (HbA1c) ≥ 8% should not undergo elective hernia surgery and should be referred to an endocrinologist for intensive diabetic management.^65^ Implementing perioperative glycaemic control and maintaining perioperative plasma glucose levels below 11.1 mmol/L in both diabetic and non-diabetic patients was strongly recommended in the 2017 CDC SSI prevention guidelines.^13^ The 2016 WHO guidelines reviewed 15 RCTs of perioperative glycaemic control in adults, and found intensive protocols with strict blood glucose target levels were associated with a significant decrease in SSI incidence compared with conventional protocols (OR 0.43; 95% CI 0.29–0.64).^68^

## Use of Drains in Hernia Surgery

Closed suction drains (CSD) are often used in open VHR in an attempt to control dead space and prevent postoperative haematoma or seroma formation, which have been implicated in SSI.^69^ Seromas are more common after mesh onlay than mesh sublay/retrorectus repair, due to the creation of lipocutaneous flaps in mesh onlay repairs.^69^ However, the clinical relevance of seromas has been questioned, unless persisting after 6 months or symptomatic. Most seromas have a peak formation at 2 weeks after hernia surgery, when drains have usually been removed. There is a lack of high-quality evidence that subcutaneous CSD reduce seroma formation, surgical site occurrence requiring procedural intervention (SSOPI) or SSI after VHR repair with mesh.^70–73^

Drains may be a surrogate marker for more complex hernia surgery or high-risk patients, and thus be identified in univariate analyses as a risk factor for SSIs.^70–73^ Colonization of drains by skin organisms or environmental pathogens, including multi-resistant organisms, occurs after 24 h.^74,75^ Surgeons place drains in more than 50% of open VHR repairs.^69^ However, the decision to place and subsequently remove CSD in VHR is often related to individual *surgeon preference* rather than evidence-based practise, due to the paucity of RCTs.^76,77^

**Fig. 5:**
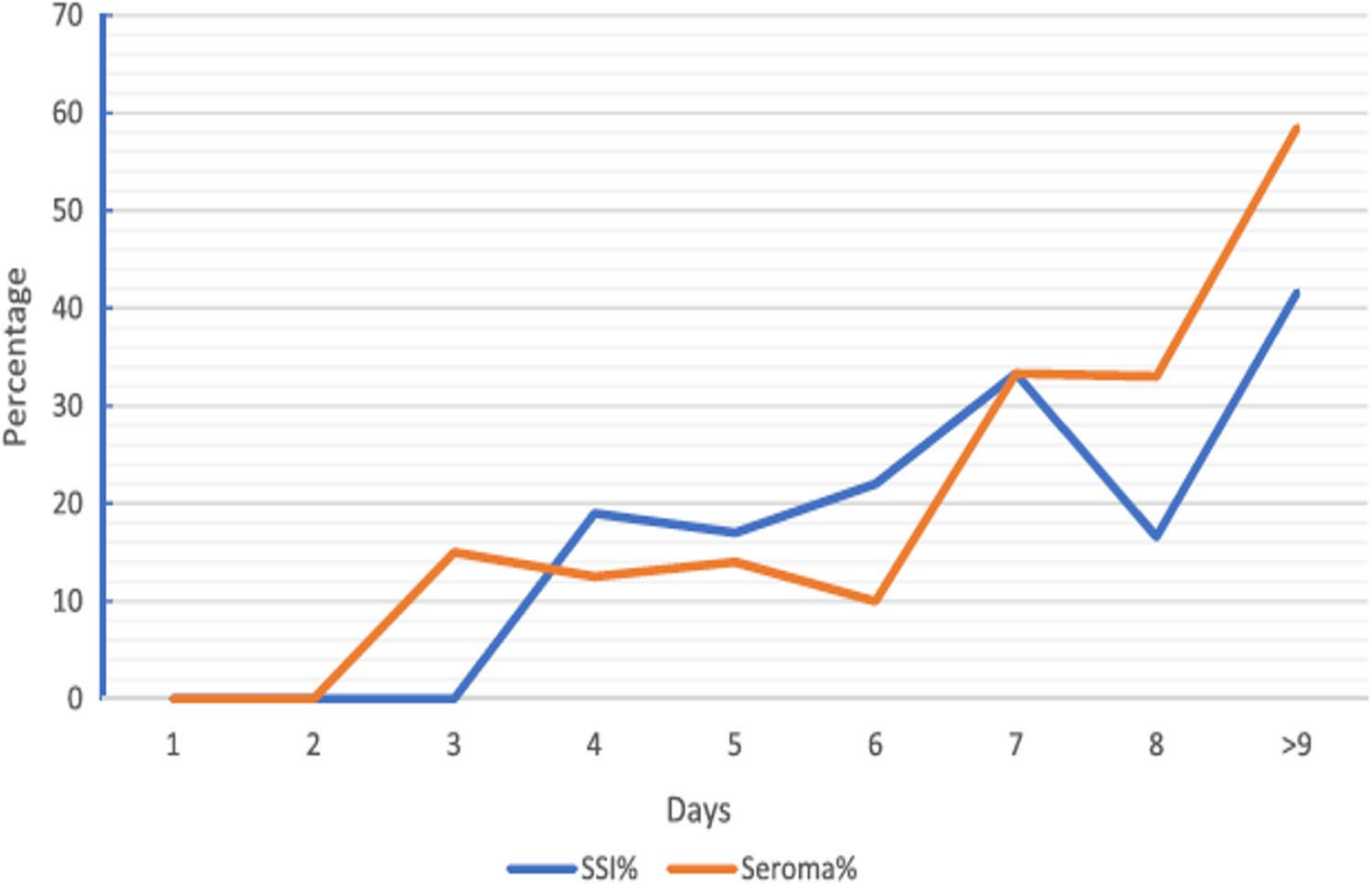
Correlation of duration of drain (in days) after elective and emergent open VHR with daily percentage of SSI (surgical site infection) and seroma formation. Reproduced by permission^80^

Retrospective studies have suggested the use of drains may be counterproductive by increasing postoperative pain, hospital length of stay (LOS) and SSI after VHR without improving seroma formation rates.^78–80^ In a retrospective analysis of 64 clean VHR, a statistically significant linear relationship was found to exist between longer duration of wound drainage and *increased* development of wound SSOs (superficial cellulitis, seroma/hematoma, superficial SSI, deep SSI), even when adjusted for obesity.^78^ Longer duration of wound drainage (> 7 days) was also found to be significantly associated with SSI and seroma formation in a retrospective series of 186 elective and emergency open VHR, reported by Idrees et al. (2021)^80^ (Fig. 5). Kushner et al. (2021) reported a retrospective series of posterior component separation with Transversus abdominus release (TAR)/retrorectus mesh placement in 184 consecutive operations comparing early drain removal (at hospital discharge) in 95 patients versus late drain removal in 89 historical control patients. The mean postoperative day ± SD of early drain removal was 5.91 ± 5.16 days, versus the late removal cohort, 16.62 ± 5.82 days (*p* < 0.01). No differences in SSO, SSI, seroma or re-admissions (all cause or for wound-related complications) were found. It was concluded that after VHR utilizing TAR, it was safe to remove all drains at hospital discharge, regardless of drain output.^71^ Ramshaw et al. (2016) studied the effect of a comprehensive clinical QI initiative begun in 2013 which included initiation of TAR/subcutaneous quilting sutures and *no* drains for VHR. They compared 33 historical control patients from 2011 to 2013 to 69 patients from 2013 to 2015. The combination of QI initiative/TAR introduction/elimination of drains resulted in lower risk of major wound complications (OR, 0.21 (95% CI = 0.05–0.88)), minor wound complications (OR, 0.24 (95% CI = 0.07–0.82)), hernia recurrence (OR, 0.05 (95% CI = 0.01–0.39)) and pulmonary complications, and a shorter hospital LOS.^73^

This *surgeon preference* of placing drains may be an extrapolation from laparotomies, where use of subcutaneous drains in high-risk patients may reduce SSIs, including patients who are obese and/or have contaminated/dirty wound types. However, there is no evidence from systematic reviews of RCTs that subcutaneous drainage in *all* patients having laparotomies reduces SSI risk, or in patients identified to have clean or clean-contaminated wounds.^81,82^ The most recent Cochrane review of subcutaneous drain use in hernia surgery was published in 2013. It reported no suitable RCTs of drain versus no drain use in open incisional hernia repair.^76^

Since this study, there is only one suitable RCT of the use of CSD in open elective VHR. This was a study of large incisional hernia repair by macroporous polypropylene mesh onlay technique in a total of 42 patients, published in 2015. Patients were randomly allocated to subcutaneous drainage or suturing of the subcutaneous fat to the aponeurosis with a quilting technique using 2–0 polyglactin 910 sutures. Drains were removed when drainage was < 40 ml/24 h. There was a high overall incidence of seroma (52.4%). Most of these had resolved at 90-day follow-up, with only 23% requiring intervention. No difference in seroma or SSI incidence was found between the two groups.^70^ Weiss et al. (2019) conducted a systematic review of studies involving prolonged prophylactic antibiotic (PPA) “coverage” of CSD in VHR and rates of SSI. Five studies were suitable, involving a total of 772 patients. They concluded PPA use could not be supported, the literature evidence of an association between CSD and SSI was limited and conflicting, and RCTs are required to determine if CSD actually promote or prevent SSI in VHR.^72^ The impetus for performing such RCTs may have diminished in clean elective VHR surgery due to the evolution of minimally invasive VHR with fascial closure, and use of posterior component separation/TAR in open VHR. However, from the NSQIP data of 10-year hernia repair trends, in 2017, only 36.6% of VHR were performed by a minimally invasive approach. Thus, the question of CSD use in open VHR is still relevant, not only in the USA but also the global surgical community.^83^

Guidelines do not recommend routine use of drains in clean surgery, but if one is used, it should be removed early to prevent bacterial contamination and shorten LOS.^84^ Extended antibiotic coverage for wound drains in clean and clean-contaminated wounds should not be used.^13,68^ The WHO guidelines recommended against using antibiotic incisional wound irrigation before wound closure to prevent SSI.^68^ There were no existing RCTs which evaluated soaking prosthetic devices in antimicrobial solutions before implantation in humans for the prevention of SSI.^13,68^ Some animal trials and retrospective clinical trials in human subjects reported significantly improved outcomes with antibiotic pre-soaking/irrigation of hernia mesh, particularly in fields contaminated with MRSA or enteric organisms.^9,11^ In an animal study of vancomycin pre-soaking followed by MRSA inoculation of different types of macroporous mesh, the hydrophilic polyester meshes and hydrogel composite polypropylene mesh had greater uptake of vancomycin antibiotic than non-composite hydrophobic polypropylene mesh. This resulted in improved MRSA bacterial clearance after inoculation in these meshes versus *zero* clearance in untreated polyester. However, untreated polypropylene was more resistant to infection and showed less biofilm formation than polyester mesh, with both the vancomycin-treated composite and non-composite polypropylene meshes having no MRSA biofilm on SEM.^11^

Use of prophylactic negative pressure wound therapy (pNPWT) on clean, primarily closed surgical incisions in high-risk conditions for prevention of SSIs was recommended in the 2016 WHO guidelines.^68^ This may be an alternative to suction drains after hernia mesh repair surgery. Prophylactic NPWT (PICO; Smith & Nephew, London, UK) compared to conventional dressing (MEPORE pro; Molnlycke, Goteborg, Sweden) reduced the SSI rate from 8 to 0% (*p* < 0.002) and overall SSOs from 29.8 to 16.6% (*p* < 0.042) in a RCT involving 150 patients having open VHR using Rives-Stoppa repair, TAR or anterior component separation. It was concluded pNPWT should be used in high-risk wounds or obese patients undergoing open VHR, or when anterior compartment separation is used.^85^

## ACS-NSQIP Surgical Risk Calculator

The universal ACS-NSQIP Surgical Risk Calculator allows surgeons to provide an immediate prediction of an individual patient’s operative risk (SSI, major complication, death) and outcomes (LOS, re-admission, discharge to nursing or rehabilitation facility) by factoring in the surgical procedure and patient comorbidities. The predicted outcome profile can be presented to the patient and their families in a patient-friendly format, and enable proper informed consent to be obtained. It may also provide important perspectives as to whether the operative risk is prohibitive or whether the operation should be postponed and the risk improved.^86^ In the case of elective VHR, there is opportunity for pre-operative modification of comorbidities including obesity, tobacco smoking, diabetes mellitus and COPD by weight loss programmes, smoking cessation, diabetic control, nutritional support, tailored exercise and vitamin supplementation. Such simple principles of prehabilitation should be integrated into the overall care-plan for surgical patients. This can improve surgical outcomes; minimize SSIs, major complications and healthcare-related costs; and facilitate return to normal activities. Such outcomes have been achieved in both complex and routine VHR by proper implementation of evidence-based QI interventions without major cost burdens to the hospital system.^14^

## Conclusions

Mesh infection is a highly morbid complication after hernia surgery, and is associated with hospital re-admission, increased healthcare costs, re-operation, hernia recurrence, impaired quality of life and plaintiff litigation. Implementation of perioperative SSI prevention “bundles” based on international and national guidelines can potentially prevent up to half of all SSIs. The ACS-NSQIP and AHSQC registries provide outcomes after hernia surgery from very large datasets of patients. Those patients with large, complex ventral herniae requiring open surgery who have multiple comorbidities (advanced age, ASA score ≥ 3, malnutrition, diabetes, immunosuppression, tobacco smoking or obesity (BMI ≥ 35 kg/m^2^)) are particularly at risk of SSIs. Pre-operative patient optimization includes weight loss, cessation of smoking and control of diabetes. Intraoperative interventions relate, in particular, to control of fomite mediated transmission in the operating theatre and prevention of mesh contamination with *S. aureus* CFUs*.* Risk management strategies should also target the *niche ecological conditions* which enable bacterial survival and subsequent biofilm formation on an implanted mesh. These include hyperglycaemia, hypoxia, hypothermia, hypoperfusion, hypovitaminosis, haematoma, large lipocutaneous flaps, inadequate tissue levels of prophylactic antibiotics and microporous mesh. Outcomes of mesh infection after hernia surgery are closely related to mesh type and porosity, patient smoking status, presence of MRSA, bacterial adhesion and biofilm production. The use of macroporous polypropylene versus absorbable biosynthetic P-4HB mesh or biological mesh in contaminated wounds requires further RCTs. Suction drains may be a surrogate marker for more complex hernia surgery or high-risk patients. There is a paucity of evidence that CSD prevent SSIs after hernia surgery. Prophylactic NPWT may provide an alternative to CSD in high-risk wounds after VHR. The utility of the ACS-NSQIP Surgical Risk Calculator in predicting complications and outcomes in individual patients, and the importance of QI initiatives in surgical units is emphasized.
