# Correction to: Risks and Prevention of Surgical Site Infection After Hernia Mesh Repair and the Predictive Utility of ACS-NSQIP

**DOI:** 10.1007/s11605-022-05550-3

**Published:** 2022-12-05

**Authors:** Robert Beaumont Wilson, Yasser Farooque

**Affiliations:** Department of Upper Gastro-Intestinal Surgery, Liverpool Hospital, UNSW, Sydney, NSW 2170 Australia

**Correction to: Journal of Gastrointestinal Surgery (2022) 26:950–964**

**https://doi.org/10.1007/s11605-022-05248-6**

This article has been updated with correction.

